# Analytical Validation of Esopredict, an Epigenetic Prognostic Assay for Patients with Barrett’s Esophagus

**DOI:** 10.3390/diagnostics14182003

**Published:** 2024-09-10

**Authors:** Sarah Laun, Francia Pierre, Suji Kim, Daniel Lunz, Tara Maddala, Jerome V. Braun, Stephen J. Meltzer, Lisa Kann

**Affiliations:** 1 Previse, Halethorpe, MD 21227, USA; 2 Division of Gastroenterology and Hepatology, Department of Medicine, Johns Hopkins University School of Medicine, Baltimore, MD 21287, USA

**Keywords:** EAC, HGD, Barrett’s esophagus (BE), prognostic assay

## Abstract

Esopredict^TM^ is a prognostic assay that risk-stratifies Barrett’s esophagus patients to predict future progression to high-grade dysplasia (HGD) or esophageal adenocarcinoma (EAC). Established based on foundational studies at Johns Hopkins University, a risk algorithm was developed and clinically validated in two independent studies (*n* = 320). Esopredict^TM^ is currently offered as a clinical test under the Clinical Laboratory Improvement Amendments (CLIA) guidelines. Here we present the analytical validation by repeated testing of FFPE tissues (*n* = 26 patients), cell lines, and contrived DNA controls to determine assay performance regarding analytical sensitivity (as defined by the limit of detection (LOD)), analytical specificity (as defined by the limit of blank (LOB)), accuracy as determined from the average positive and negative agreement, repeatability, and reproducibility. The LOD for the assay at 1.5% DNA methylation was significantly higher than the LOB, as determined by an unmethylated DNA control (0% methylated DNA). Inter- and intra-assay average positive agreement (APA) were 88% and 94%, respectively, while average negative agreement (ANA) values were 90% and 94%, respectively. Average inter- and intra-assay precision were <9% and <5% coefficient of variation (CV), respectively. These results confirm that Esopredict^TM^ is a highly reproducible, sensitive, and specific risk categorization assay for the prediction of progression to HGD or EAC within 5 years.

## 1. Introduction

Barrett’s esophagus (BE) is characterized by the replacement of the normal squamous epithelial cells lining the esophagus with intestinal metaplastic tissue, resulting in a visibly reddened and thickened appearance. Gastrointestinal reflux disease (GERD), marked by the retrograde flow of stomach acid into the esophagus, leading to chronic inflammation and irritation, is a major risk factor for BE [1,2,3]. BE is most commonly diagnosed in patients over 50, with an average diagnosis at 55 years, and exhibits a higher occurrence among males compared to females [1,4,5,6]. Other risk factors include smoking, alcohol use, obesity, and family history [7,8,9,10,11].

Patients diagnosed with BE are at a 10–55× risk of developing esophageal adenocarcinoma (EAC), which is among the most lethal cancers, with a 21% 5-year survival rate [1,6,12,13]. Due to the increased risk and the lethality of EAC, the American College of Gastroenterology guidelines suggest that patients with BE be enrolled into endoscopic surveillance programs with the goal of detecting dysplasia or early cancer, both of which can be treated effectively with endoscopic eradication therapy (EET) [14]. However, the detection of dysplasia is fraught with challenges, including interobserver variability and subjectivity, establishing the need for better risk stratification tools [15,16,17].

Methylation biomarkers have been validated as methods for earlier detection and prognostication of cancers and their precursor conditions, including BE [18,19,20,21,22]. In background studies performed at Johns Hopkins University and validation studies at Previse, four specific methylation markers (*p16*, *RUNX3*, *HPP1*, and *FBN1*) have demonstrated consistent statistically significant performance as early harbingers of neoplastic progression in BE, presenting an opportunity for improved prognostication in BE patients [23,24,25,26].

Esopredict^TM^ is a high-complexity, laboratory-developed test (LDT) developed at Previse (Baltimore, MD; CLIA #21D2256153). This tissue test (FFPE) is indicated for BE patients with non-dysplastic BE (NDBE), indefinite for dysplasia (IND), or low-grade dysplasia (LGD) to predict future progression to high-grade dysplasia (HGD) or esophageal adenocarcinoma (EAC). This epigenetic test, Esopredict^TM^, has been clinically validated in two independent studies: a 110-patient study conducted across six clinical sites, and 224 samples across 80 patients in a spatial/temporal cohort [25]. These studies have demonstrated the robustness of Esopredict^TM^’s performance to identify a subset of patients who are at either higher or lower risk than average of advancing to HGD or EAC to help guide treatment decisions by physicians through either increased surveillance or decreased surveillance, respectively.

While clinical validation is vital to establish the accuracy of the test with respect to a clinical outcome, analytical validation is equally essential to establish the accuracy and reliability in measuring the signal across analytical conditions. The CLIA ’88 guidelines and Clinical Laboratory Standards Institute (CLSI) provide well-accepted guidance for analytical validation for nucleic acid assays, including reproducibility and repeatability, sensitivity, and specificity. In this study, we aimed to demonstrate the robustness of Esopredict^TM^ within acceptable ranges across multiple conditions (e.g., reagents, instruments, runs, operators), through a comprehensive analysis of samples from 26 patients with BE, cell lines, and methylated and unmethylated controls. We aim to show through rigorous analysis that Esopredict^TM^ is an assay with high precision, accuracy, analytical sensitivity (as measured by the limit of detection (LOD)) and analytical specificity (as measured by the limit of blank (LOB)) for the determination of the risk of progression to HGD or EAC.

## 2. Materials and Methods

### 2.1. Test Overview

Esopredict^TM^ is a CLIA-developed LDT for high-complexity analysis of methylation markers in tissue from Barrett’s esophagus patients for predicting the risk of future progression to EAC or HGD within five years. The test was developed from a series of studies performed at the Johns Hopkins School of Medicine, Baltimore, MD, USA and validated in the CLIA Laboratory at Previse in Baltimore, MD, USA [23,24,25]. DNA is extracted from FFPE tissue of esophageal origin in Barrett’s patients. Following bisulfite conversion, methylation-specific PCR (MSP) is performed to assess methylation levels of four genes (*HPP1*, *p16*, *RUNX3*, and *FBN1*) as well as a control gene *beta*-actin. Normalized methylation values and age are used to generate a risk score and risk category (low vs. high) as well as the probability of progression within five years. The test is intended for patients > 18 years.

### 2.2. Validation Samples

A total of 26 samples were evaluated including FFPE patient specimens, cell lines, DNA and contrived controls (see Appendix A). Tissue Samples: Sixteen Barrett’s esophagus patient samples were de-identified and submitted from collaborating sites. Serial sections were cut from formalin-fixed paraffin-embedded (FFPE) tissue blocks. Two additional esophageal tissue samples were purchased from Biochain Institute Inc., Newark, CA, USA (C509126, human normal esophagus) and Tissue Array, Derwood, MD, USA (HuCat041, human EAC esophagus).

Cell lines: Three EAC-derived cell lines from unique patients were obtained, including two human esophageal cancer cell lines from Sigma Aldrich Burlington, MA, USA (FLO-1, SK-GT-4) and a proprietary esophageal cell line, JH-EsoAd1, from Dr. Stephen J. Meltzer’s laboratory at the Johns Hopkins School of Medicine, Baltimore, MD, USA [27].

DNAs: Fully methylated-100% methylated DNA, (HCT116 DKO Methylated DNA, D5014-2), and unmethylated DNA-0% methylated DNA (Human HCT116 DKO Non-Methylated DNA, D5014-1) were obtained from Zymo Research, Irvine, CA, USA. The 100% methylated sample was used as a positive assay control and the 0% methylated sample was used as a negative assay control for Esopredict^TM^.

Contrived samples were engineered for limit-of-detection (LOD) analysis using the fully methylated DNA (100% methylated) in a background unmethylated DNA (0% methylated) by mixing DNA from both methylated and unmethylated samples at compositions of 5%, 3%, and 1.5% methylated DNA. The fully unmethylated DNA was used as the limit of blank (LOB).

We note that the cell lines, DNA and contrived samples are not derived from FFPE, which is the source material for Esopredict^TM^. Given the difficulty in finding an unlimited FFPE source of sufficient quality and quantity for this study, we feel the cell lines and DNA provide an appropriate surrogate for assessing assay performance. Additionally, amplicons were designed to accommodate the fragmented nature of FFPE samples with amplicons < 200 base pairs. FFPE specimens were also selected across a range of Esoscores and DNA input levels.

Ethical statement for human subjects: Histological tissues from patients diagnosed with Barrett’s were prepared at the time of endoscopy and not collected for this study. Archival tissues were anonymized and de-identified. The study was exempted by the institutional ethics committee of Johns Hopkins University, Baltimore, MD, USA.

### 2.3. Test Method

DNA was extracted from anonymized FFPE tissue samples from BE patients using the Qiagen, Germantown, MD, USA QIAamp DNA FFPE Advanced Kit (56604). DNA was extracted from cell lines using the Qiagen DNeasy Blood and Tissue Kit (69504). DNA was quantified using the Qubit 4.0 and Qubit ds high-sensitivity kit (Q32854). For bisulfite treatment, 100–500 ng of DNA was used in the Zymo, Irvine, CA, USA Research EZ DNA Methylation-Lightning Kit (D5030T), which is within the range of the kit and where 100 ng is the minimal amount for Esopredict^TM^. 

Methylation-specific PCR was performed on the Quantstudio 3 software version 1.5.2 (ThermoFisher, Carlsbad, CA, USA) using a set of 5 sets of primers and probes (IDT) for four methylation genes (*HPP1*, *p16*, *RUNX3*, and *FBN1*) as well as a control gene *beta*-actin (see Appendix B). All probes were labeled with a universal quencher and FAM. A five-point standard curve was made by dilution of a 100% methylated control from Zymo Research, Irvine, CA, USA (D5015). Fivefold dilutions were constructed in the range of 50 ng DNA to 0.08 ng DNA. Briefly, 20 µL reactions were set up consisting of 3–17 ng DNA, 0.5 µM forward and reverse primers, 0.25 µM probe, and TaqMan Fast Advanced Master Mix (no UNG) (ThermoFisher, Carlsbad, CA, USA, A44360). Cycling conditions were 95 °C 5 min, followed by 40 cycles of 95 °C 0:15, 60 °C 0:25, and 72 °C 0:30. Each sample was run in triplicate, and quantities were averaged. Samples where amplification was detected but below the assay limit of quantification or lower than the lowest standard of 0.08 ng DNA were imputed to LLOQ/2 (0.04 ng). Each gene was analyzed, and normalized methylation values (NMV) were obtained for all methylation genes by dividing by control gene beta-actin:

NMV = Methylation gene/*beta*-actin × (100)

### 2.4. Data Analysis

Using the normalized methylation values combined with age at biopsy, a classification algorithm was developed to calculate an Esoscore on a scale of 0–100, which translates into four risk levels for progression to HGD or EAC within 5 years: low risk, low-moderate risk, high-moderate risk and high risk, as shown in Table 1. In summary, the algorithm transforms NMV values for each gene and uses a linear regression model with age to determine the Esoscore. Given the high correlation between *HPP1* and *FBN1*, an equally weighted average combines these two markers for analysis. The score for a BE patient with non-dysplastic BE (NDBE), indeterminate dysplasia (IND), or low-grade dysplasia (LGD) is translated into a probability of progressing to HGD or EAC within 5 years (see Appendix C). Note: For Esopredict^TM^ calculations for contrived DNA samples and cell lines, we used a patient age of 55 years, which is the average age of diagnosis for Barrett’s esophagus [4,5,6].

### 2.5. Assay Performance Characteristics

#### 2.5.1. Limit of Blank (LOB)

LOB was determined by calculating the Esoscore for the unmethylated DNA sample (0% methylation). Seventy-five independent preps of the unmethylated sample were analyzed across 66 runs. The LOB was determined as the highest value likely to be observed within confidence limits for the unmethylated material. Non-template controls or water blanks were also analyzed in each assay but not used in LOB analysis since there was no amplification detected in any genes, including the control gene; therefore, no Esoscore can be determined. LOB was calculated using the formula:

LOB = mean_unmethyated_ + 1.645(SD_unmethylated_)

#### 2.5.2. Limit of Detection (LOD)

To determine the assay, LOD 100% methylated DNA was mixed in a background of unmethylated DNA at various low-level concentrations to determine the lowest analyte concentration to be reliably distinguished from the LOB. A 5%, 3%, and 1.5% methylated sample was analyzed using the assay minimum input of 100 ng DNA for the bisulfite reaction and 3.5 ng per qPCR. Contrived samples were analyzed across four runs with an average of 5 replicates per run. LOD was determined using the formula:

LOD = LOB + 1.645(SD_Low concentration sample_)

#### 2.5.3. Accuracy, Precision (Reproducibility and Repeatability)

The average agreement between sample measurements was determined by repeating 18 BE patient samples five times across three runs (two runs in singlicate, one run in triplicate) on different days using different equipment, operators, and reagents. Due to the limitation of DNA extracted from FFPE samples and the quantification limitations of the assay, triplicates could only be performed in one run for patient samples. Cell lines were also run in triplicate across three independent runs to compensate for the lack of tissue material. Two of the three cell lines were analyzed in triplicate at the highest and lowest input levels (100 ng and 500 ng). As there is no ground truth or gold standard when comparing replicates, ANA (average negative agreement) and APA (average positive agreement) were calculated. APA is the number of higher-risk level matches as a proportion of the total number of higher-risk results observed, and ANA is the number of lower-risk level matches as a proportion of the total number of lower-risk results observed as calculated in Table 2 [28]:

R1 = Replicate 1 and R2 = Replicate 2 APA = 2A/(2A + B + C) ANA = 2D/(2D + B + C)

We also include the distinction between the two main risk categories (low vs. high risk) as those distinguish populations with a probability of progressing to HGD or EAC less than the average BE population versus those who have a probability of progressing greater than the average BE population [25] and represent potential reduced vs. increased esophagogastroduodenoscopy surveillance populations. During initial model development, risk levels were further refined to include four clinically relevant actionable risk levels, thereby creating a more substantial impact on decisions [25].

Additionally, intra-assay and inter-assay precision were determined by calculating the coefficients of variation (CV) for the Esoscore for all sample types and patient samples and across all risk levels.

## 3. Results

### 3.1. Limit of Blank (LOB)

There was no amplification detected in any of the methylation genes in the unmethylated DNA except p16, where there was low-level amplification in >80% of the unmethylated samples (NMV < 1%) and only one sample (1% of total) had very low amplification for FBN1 (NMV < 0.1%). LOB and LODs were calculated using the formulas:

LOB = mean_unmethyated DNA_ + 1.645(SD_unmethylated DNA_) LOD = LOB + 1.645(SD_1.5%DNA_)

LOB values are shown relative to the LOD as normalized methylation values in Table 3 and for the Esosore in Table 4.

### 3.2. Limit of Detection (LOD)

Contrived samples were constructed at 1.5%, 3%, and 5% methylated DNA by mixing 100% methylated DNA in a background of unmethylated DNA (0% methylated) to determine the lowest methylation values distinguished from unmethylated DNA. The LOD level was determined as the lowest methylation level, which can be reliably distinguished from the LOB or fully unmethylated sample. 

**Figure 1:**
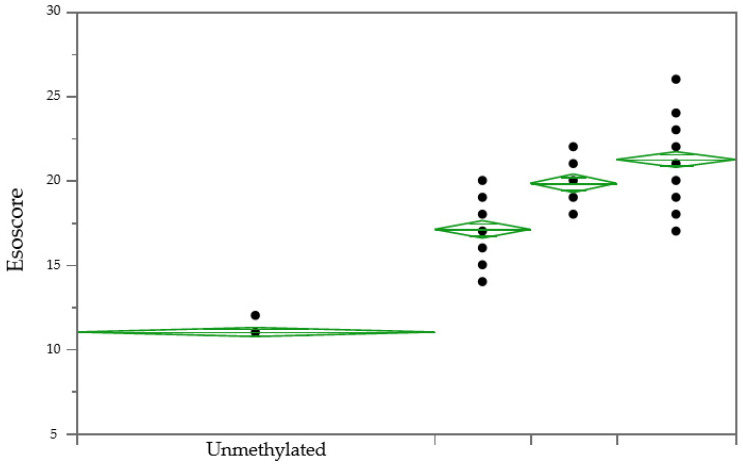
Esoscore vs. % Methylated DNA Oneway ANOVA (Prob > F, <0.0001). Samples are unmethylated DNA, 1.5% fully methylated DNA (1.5% FM), 3% fully methylated DNA (3% FM) and 5% fully methylated DNA (5% FM). Note that each dot may represent multiple data points.

An ANOVA analysis of Esoscores demonstrated that all levels were statistically distinguishable (Figure 1, Table 5 (Prob > F, <0.0001)) and the 1.5% methylation was determined as the LOD. Additionally, pairwise comparisons confirmed that each level is statistically distinguishable, and a *t* test < 0.01 demonstrates that differences in 1–2% methylation levels are detectable.

Although the LOD analysis was performed on non-FFPE source material and FFPE may pose limitations due to quantity and quality, we did not observe issues with the FFPE samples in the study due to the strong correlation of the control gene beta-actin values and DNA input (F < 0.0001 for both FFPE and non-FFPE sources). Additionally, FFPE samples were included across all risk levels and DNA inputs and performance were similar to control samples. 

### 3.3. Accuracy, Precision (Repeatability and Reproducibility)

**Figure 2:**
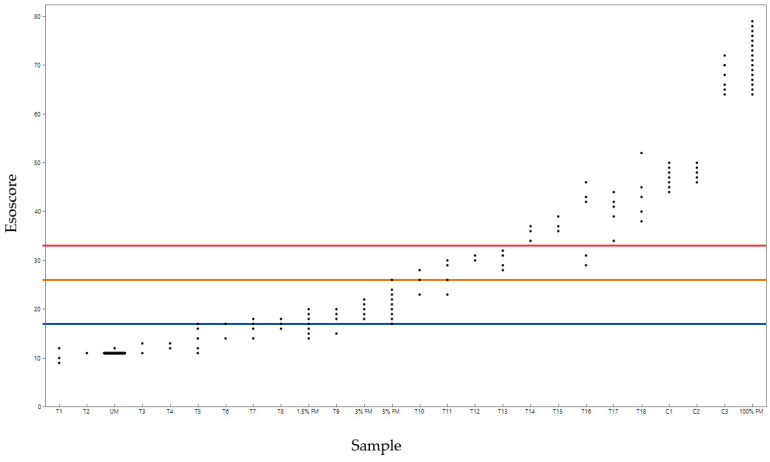
Esoscore results for all samples in the study. The horizontal orange line at 26 represents the boundary of lower-risk levels (low, low moderate) to higher-risk levels (high-moderate, high). T (tissue), C (cell line), UM (unmethylated DNA), 1.5%FM (1.5% fully methylated DNA), 3%FM (3% fully methylated DNA), 5%FM (5% fully methylated DNA), and 100%FM (100% fully methylated DNA). Blue line is the cutoff for low to low-moderate risk, orange is low-moderate to high-moderate risk level and red is high-moderate to high-risk level. Note: for the UM sample multiple samples are represented at Esoscore 10 indicated by the black line.

Esoscore results for all 26 samples are presented in Figure 2. 

APA was calculated as a sample agreement within the higher-risk levels, and ANA was a sample agreement within lower-risk levels. Inter- (within) and intra- (between) assay results are shown in Table 6 for comparison of all risk levels in addition to the higher versus lower-risk categories. Discordant samples were samples that bridged cutoff boundaries for risk levels (see Figure 2, e.g., Samples T10, T11). Note: Intra-assay was only performed on 17 of the 18 tissue samples due to the limitation of material for T2. In no instance did a sample with Esoscores from the lowest risk have samples also in a higher-risk level and vice versa (samples tested in the highest risk level also did not exhibit replicates or repeat tests in the lower-risk levels), and samples occurring in multiple risk levels were those near cutoff boundaries.

**Figure 3:**
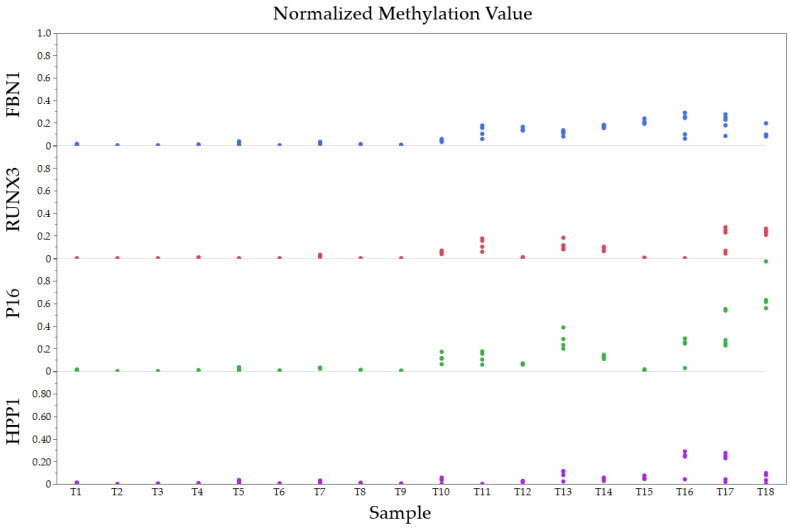
Normalized methylation values (NMV) for each gene and tissue sample.

**Figure 4:**
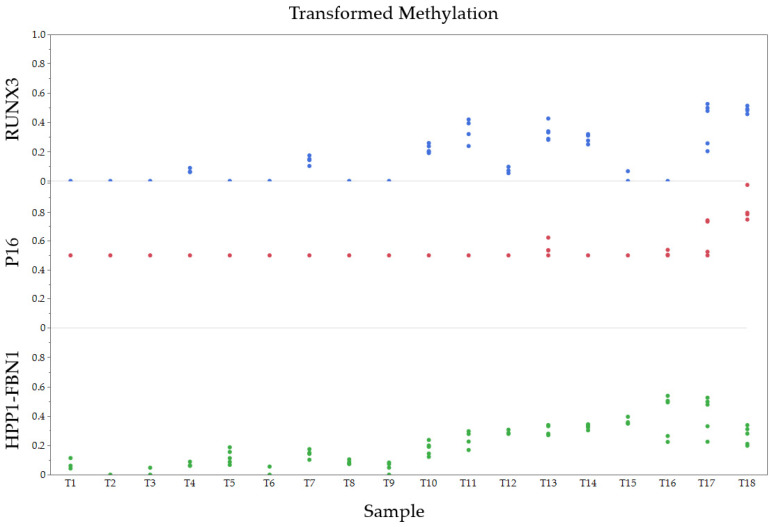
Transformed normalized methylation values for each gene and tissue sample.

The normalized methylation values for each gene and transformed values for patient tissue samples are shown in Figure 3 and Figure 4. 

The transformed values are utilized in the model to generate the Esoscore. The Esoscore variability between (inter) and within (intra) runs was evaluated for the 18 patient samples in each risk level and represented as %CV (coefficient of variation), as shown in Table 7. Data for all 26 samples in the study are represented in Table 8. 

## 4. Discussion

Esophageal cancer is the second most lethal cancer in the United States with a 5-year survival of <21% and is amongst the most rapidly rising cancers today, with a more than 7-fold incidence increase over the past few decades [2,29,30,31,32]. Thus, patients diagnosed with EAC’s precursor, Barrett’s esophagus, are enrolled in endoscopic surveillance programs. Historically, such risk stratification has been a challenge, with surveillance decisions primarily being determined by the presence of dysplasia (or lack thereof). However, there is high interobserver variability amongst pathologists in diagnosing dysplasia, and diagnosis is not a great predictor of future neoplastic progression [15,16,17]. Further, in patients with non-dysplastic Barrett’s esophagus, from which 50% of esophageal adenocarcinoma cases arise, gastroenterologists lack the tools to identify which patients may or may not progress. For surveillance programs to be successful, methods of improving risk-stratification are needed to determine how often to administer endoscopy, and potentially, whom to administer endoscopic eradication therapy. 

Esopredict^TM^ is a proprietary epigenetic laboratory test to predict 5-year risk of progression to high-grade dysplasia or esophageal adenocarcinoma in patients with Barrett’s esophagus. This solution has been clinically validated, and its performance could lead to better treatment options such as more or less frequent surveillance, or increased surveillance or EET, based on risk categorization. It uses available biopsy material from endoscopic exams, so no additional biopsy material is needed from the patient and can be run using standard molecular laboratory equipment and techniques.

In this analytical validation study, we have demonstrated that Esopredict^TM^ has high analytical sensitivity, analytical specificity, and reproducibility for the intended population. Analytical specificity and sensitivity were measured by LOB and LOD analyses where as low as 1.5% methylation composition may be detected from a background of unmethylated DNA. Furthermore, 1–2% methylation differences may be detectable. Given that Esopredict^TM^ scores are also age-dependent, it is worth noting that very old patients (>95-year-old) will rarely have a risk level lower than high-moderate due to the LOD for that age. However, the average patient age is 55 years [1,4,5,6] and an older age group >95 years is unlikely to receive testing or surveillance. The test was also highly reproducible across a range of Esoscores that span all risk categories with an average CV < 10%. High reproducibility was also demonstrated by ANA (>90%) and APA (>88%) analysis. As Esopredict^TM^ is reported as a continuous result with an associated probability of HGD or EAC and is not reported solely as dichotomous (e.g., positive or negative), it is not surprising that samples near the cutoff in the intermediate range (low-intermediate and high-intermediate) will shift upon repeat testing. It is also worth noting that all repeats in the lowest risk category scored low risk (i.e., risk levels low or low-moderate) and all repeats in the highest risk category scored high risk (i.e., risk levels high-moderate or high). This analytical validation study demonstrates that Esopredict^TM^ is a robust and reproducible assay. 

## 5. Conclusions

We demonstrate that the prognostic test Esopredict^TM^ performs to the expectations of such accrediting bodies as CMS CLIA with high repeatability and reproducibility and with high analytical sensitivity and specificity, as shown by the data presented here. This assay shows a limit of detection of 1.5% methylation in samples with a minimal DNA input of 100 ng. Overall inter and intra-assay precision for Esoscores demonstrated high reproducibility over a range of risk levels CV < 9% and <5%, respectively, and are within recommendations for clinical assays according to CLIA/CLSI guidelines (CV < 20%). Inter and intra-assay average positive agreement (APA) demonstrates high concordance of 88% and 94%, respectively, in addition to average negative agreement (ANA) values of 90% and 94%, respectively, for distinguishing low-risk from high-risk populations.

## 6. Patents

Laun, S.; Kann, L.; Lunz, D.G.; Meltzer, S.J. Methods for predicting progression of Barrett’s esophagus. Patent Application US 027724, 2024

## Figures and Tables

**Table 1:** Esoscore summary for scoring, risk level, and probability of progression to HGD or EAC in 5 years [25].

Esoscore	Risk Level	5 Year Probability of Progression
0–16	Low	≤2.8%
17–25	Low Moderate	>2.8–6.2%
26–32	High Moderate	>6.2–11.2%
33–100	High	>11.2%

**Table 2:** Replicates for ANA and APA Esoscore level comparisons where R1 = Replicate 1 and R2 = Replicate 2 and High refers to higher risk level and Low to lower risk level.

	R1 High	R1 Low
R2 High	A	C
R2 Low	B	D

**Table 3:** Normalized methylation values (NMV) for each gene at the LOB and LOD.

	Normalized Methylation Value	
Gene	LOB	LOD
*HPP1*	0.0%	1.4%
*p16*	0.6%	1.6%
*RUNX3*	0.0%	1.7%
*FBN1*	0.0%	1.2%

**Table 4:** Esoscore limit of blank (LOB) and limit of detection (LOD) for a 55-year-old patient (i.e., average age of BE patient).

	Esoscore	
	LOB	LOD
Esoscore (55 years)	11	14

Note: Given that the Esoscore is age-dependent, the LOB and LOD will vary depending on age. For a very young (18 years) vs. very old (95 years) patient, LOB will be 0 and 23, respectively, and LOD 3 and 26, respectively.

**Table 5:** Average Esoscore for low-level methylated DNA with upper and lower 95% confidence intervals (CI) and inter and intra-assay %CV based on a 55-year-old patient (i.e., average age of BE patient).

Sample	Ave Esoscore	Lower 95%	Upper 95%	%CV-Inter	%CV-Intra
0% Unmethylated (LOB)	11.0	11.0	11.0	1.0%	0.0%
1.5% Methylated (LOD)	17.1	16.3	17.9	9.7%	8.3%
3% Methylated	19.8	19.2	20.5	6.3%	6.0%
5% Methylated	21.2	20.4	22.1	9.8%	6.8%

**Table 6:** Average positive and negative percent agreement within and between runs and risk levels.

Risk Levels	Inter-Assay APA	Inter-Assay ANA	Intra-Assay APA	Intra-Assay ANA
Low–Low-Moderate	44%	62%	33%	67%
Low–High-Moderate	100%	100%	100%	100%
Low–High	100%	100%	100%	100%
Low-Moderate–High-Moderate	75%	83%	86%	89%
Low-Moderate–High	100%	100%	100%	100%
High-Moderate–High	89%	89%	100%	100%
**Low/Low-Moderate–High/High-Moderate**	**88%**	**90%**	**94%**	**94%**

**Table 7:** Precision as demonstrated by inter- and intra-assay% CV across each risk category for 18 patient samples.

Risk Category	*N*	Ave Esoscore	Ave Esoscore %CV-Inter	Ave Esoscore %CV-Intra
Low	7	13	8.5%	4.0%
Low Moderate	3	20	9.1%	7.2%
High Moderate	3	29	5.9%	4.4%
High	5	39	9.7%	4.1%
		**ALL**	8.5%	4.7%

**Table 8:** Precision as demonstrated by inter- and intra-assay CV across each risk category for all 26 samples.

Risk Category	*N*	Ave Esoscore	Ave Esoscore %CV-Inter	Ave Esoscore %CV-Intra
Low	8	13	7.6%	2.7%
Low Moderate	6	20	8.8%	7.0%
High Moderate	3	29	5.9%	4.4%
High	9	48	7.1%	2.8%
		**ALL**	7.5%	4.1%

## Data Availability

The data generated in this study are not publicly available.

## Appendix A

**Table A1:** Sample details and run information.

Sample Source	Sample Type	Vendor	Total Samples Analyzed (*N* = 347)	Total Runs	Intra Assay Runs	Intra Assay Replicates
0% Methylated DNA	HCT116 DKO Cell Line DNA (methyltraferase knockout)	Zymo	75	66	3	3
1.5% Methylated DNA	Mix HCT116 UM,FM	Zymo mix	20	4	4	5
3% Methylated DNA	Mix HCT116 UM,FM	Zymo mix	18	4	4	3–5
5% Methylated DNA	Mix HCT116 UM,FM	Zymo mix	25	4	4	5–10
100% Methylated DNA	HCT116 DKO Cell Line enzymatically methylated DNA	Zymo	75	66	3	3
C1	SK-GT 4 Cell Line	Sigma-Aldrich	18	3	3	3 × 100 ng, 3 × 500 ng
C2	JHU Cell Line	JHU	18	3	3	3 × 100 ng, 3 × 500 ng
C3	FLO-1 Cell Line	Sigma-Aldrich	9	3	3	3
T2	HUCAT041 FFPE EAC	Tissue Array	3	3	0	0
T3	C509126 FFPE esophagual	BioChain	6	4	1	3
T1	FFPE BE Patient	NA	5	3	1	3
T4	FFPE BE Patient	NA	5	3	1	3
T5	FFPE BE Patient	NA	5	3	1	3
T6	FFPE BE Patient	NA	5	3	1	3
T7	FFPE BE Patient	NA	5	3	1	3
T8	FFPE BE Patient	NA	5	3	1	3
T9	FFPE BE Patient	NA	5	3	1	3
T10	FFPE BE Patient	NA	5	3	1	3
T11	FFPE BE Patient	NA	5	3	1	3
T12	FFPE BE Patient	NA	5	3	1	3
T13	FFPE BE Patient	NA	5	3	1	3
T14	FFPE BE Patient	NA	5	3	1	3
T15	FFPE BE Patient	NA	5	3	1	3
T16	FFPE BE Patient	NA	5	3	1	3
T17	FFPE BE Patient	NA	5	3	1	3
T18	FFPE BE Patient	NA	5	3	1	3

## Appendix B

**Table A2:** Primers and probes for quantitative PCR. Probes are dual-labeled with FAM and quencher.

Name	Primer/Probe	Sequence 5′ > 3′
ACTIN	Dual-labeled probe	ACCACCACCCAACACACAATAACAAACACA
ACTIN	Forward primer	TGGTGATGGAGGAGGTTTAGTAAGT
ACTIN	Reverse primer	AACCAATAAAACCTACTCCTCCCTTAA
HPP1	Dual-labeled probe	CGTTAGTTCGGATTTCGTTTTC
HPP1	Forward primer	TTCGGAGAGACGTTATTTAGTC
HPP1	Reverse primer	GCTCGCCAAACGCTAACCCGAAT
P16	Dual-labeled probe	ACCCGACCCCGAACCGCG
P16	Forward primer	TGGAGTTTTCGGTTGATTGGTT
P16	Reverse primer	AACAACGCCCGCACCTCCT
RUNX3	Dual-labeled probe	CGTTTTGAGGTTCGGGTTTCGTCGTT
RUNX3	Forward primer	GGGTTTTGGCGAGTAGTGGTC
RUNX3	Reverse primer	ACGACCGACGCGAACG
FBN1	Dual-labeled probe	CGCGTTGGAGACGGTTGTTTCG
FBN1	Forward primer	TGCGGTTGCGAGGTTTAGATTC
FBN1	Reverse primer	CTACCGAAAAACGCGAACAACG

## Appendix C

**Table A3:** Table for Esoscore and risk level determination with % probability of progression with lower confidence limit (LCL) and upper confidence limits (UCL).

EsoPredict Score	EsoCat Risk Category	Probability	Probability LCL	Probability UCL
0	Low	0.6%	0.2%	2.3%
1	Low	0.7%	0.2%	2.4%
2	Low	0.7%	0.2%	2.5%
3	Low	0.8%	0.2%	2.6%
4	Low	0.9%	0.3%	2.7%
5	Low	1.0%	0.3%	2.8%
6	Low	1.1%	0.4%	3.0%
7	Low	1.2%	0.4%	3.1%
8	Low	1.3%	0.5%	3.2%
9	Low	1.4%	0.6%	3.4%
10	Low	1.5%	0.7%	3.5%
11	Low	1.7%	0.8%	3.7%
12	Low	1.8%	0.9%	3.9%
13	Low	2.0%	1.0%	4.0%
14	Low	2.2%	1.1%	4.2%
15	Low	2.4%	1.3%	4.5%
16	Low	2.7%	1.5%	4.7%
17	Low Moderate	2.9%	1.7%	5.0%
18	Low Moderate	3.2%	1.9%	5.2%
19	Low Moderate	3.5%	2.1%	5.6%
20	Low Moderate	3.8%	2.4%	5.9%
21	Low Moderate	4.1%	2.7%	6.3%
22	Low Moderate	4.5%	3.0%	6.8%
23	Low Moderate	5.0%	3.3%	7.4%
24	Low Moderate	5.4%	3.6%	8.0%
25	Low Moderate	5.9%	3.9%	8.8%
26	High Moderate	6.4%	4.2%	9.7%
27	High Moderate	7.0%	4.5%	10.7%
28	High Moderate	7.6%	4.8%	11.9%
29	High Moderate	8.3%	5.2%	13.2%
30	High Moderate	9.1%	5.5%	14.7%
31	High Moderate	9.9%	5.8%	16.3%
32	High Moderate	10.7%	6.1%	18.2%
33	High	11.6%	6.4%	20.3%
34	High	12.6%	6.7%	22.5%
35	High	13.7%	7.0%	25.0%
36	High	14.8%	7.3%	27.7%
37	High	16.0%	7.6%	30.5%
38	High	17.3%	8.0%	33.6%
39	High	18.7%	8.3%	36.8%
40	High	20.1%	8.7%	40.1%
41	High	21.6%	9.0%	43.5%
42	High	23.3%	9.4%	47.0%
43	High	25.0%	9.8%	50.6%
44	High	26.7%	10.1%	54.1%
45	High	28.6%	10.5%	57.7%
46	High	30.5%	10.9%	61.1%
47	High	32.5%	11.3%	64.5%
48	High	34.6%	11.8%	67.7%
49	High	36.7%	12.2%	70.8%
50	High	38.9%	12.7%	73.6%
51	High	41.1%	13.1%	76.4%
52	High	43.4%	13.6%	78.9%
53	High	45.7%	14.1%	81.2%
54	High	48.0%	14.6%	83.3%
55	High	50.3%	15.1%	85.2%
56	High	52.6%	15.6%	86.9%
57	High	54.9%	16.2%	88.5%
58	High	57.2%	16.7%	89.9%
59	High	59.4%	17.3%	91.1%
60	High	61.7%	17.9%	92.2%
61	High	63.8%	18.4%	93.2%
62	High	65.9%	19.1%	94.1%
63	High	68.0%	19.7%	94.8%
64	High	70.0%	20.3%	95.5%
65	High	71.9%	21.0%	96.1%
66	High	73.7%	21.6%	96.6%
67	High	75.5%	22.3%	97.1%
68	High	77.1%	23.0%	97.4%
69	High	78.7%	23.7%	97.8%
70	High	80.2%	24.5%	98.1%
71	High	81.7%	25.2%	98.3%
72	High	83.0%	26.0%	98.6%
73	High	84.3%	26.7%	98.7%
74	High	85.5%	27.5%	98.9%
75	High	86.6%	28.3%	99.1%
76	High	87.6%	29.1%	99.2%
77	High	88.6%	29.9%	99.3%
78	High	89.5%	30.8%	99.4%
79	High	90.3%	31.6%	99.5%
80	High	91.1%	32.5%	99.5%
81	High	91.8%	33.3%	99.6%
82	High	92.5%	34.2%	99.7%
83	High	93.1%	35.1%	99.7%
84	High	93.7%	36.0%	99.7%
85	High	94.2%	36.9%	99.8%
86	High	94.7%	37.8%	99.8%
87	High	95.2%	38.8%	99.8%
88	High	95.6%	39.7%	99.9%
89	High	95.9%	40.6%	99.9%
90	High	96.3%	41.6%	99.9%
91	High	96.6%	42.6%	99.9%
92	High	96.9%	43.5%	99.9%
93	High	97.2%	44.5%	99.9%
94	High	97.4%	45.5%	99.9%
95	High	97.6%	46.4%	99.9%
96	High	97.8%	47.4%	100.0%
97	High	98.0%	48.4%	100.0%
98	High	98.2%	49.4%	100.0%
99	High	98.4%	50.3%	100.0%
100	High	98.5%	51.3%	100.0%
